# Genome-Wide Identification of *AP2/ERF* Superfamily Genes in *Juglans mandshurica* and Expression Analysis under Cold Stress

**DOI:** 10.3390/ijms232315225

**Published:** 2022-12-03

**Authors:** Minghui Zhao, Yan Li, Xinxin Zhang, Xiangling You, Haiyang Yu, Ruixue Guo, Xiyang Zhao

**Affiliations:** 1 Jilin Provincial Key Laboratory of Tree and Grass Genetics and Breeding, College of Forestry and Grassland Science, Jilin Agricultural University, Changchun 130118, China; 2 Key Laboratory of Saline-alkali Vegetation Ecology Restoration, Ministry of Education, Northeast Forestry University, Harbin 150040, China; 3 College of Horticulture, Jilin Agricultural University, Changchun 130118, China

**Keywords:** Manchurian walnut, genome-wide analysis, *CBF*, flower buds, cold resistance, expression pattern

## Abstract

*Juglans mandshurica* has strong freezing resistance, surviving temperatures as low as −40 °C, making it an important freeze tolerant germplasm resource of the genus *Juglans*. APETALA2/ethylene responsive factor (AP2/ERF) is a plant-specific superfamily of transcription factors that regulates plant development, growth, and the response to biotic and abiotic stress. In this study, phylogenetic analysis was used to identify 184 *AP2/ERF* genes in the *J. mandshurica* genome, which were classified into five subfamilies (*JmAP2*, *JmRAV*, *JmSoloist*, *JmDREB*, and *JmERF*). A significant amount of discordance was observed in the 184 *AP2/ERF* genes distribution of *J. mandshurica* throughout its 16 chromosomes. Duplication was found in 14 tandem and 122 segmental gene pairs, which indicated that duplications may be the main reason for *JmAP2/ERF* family expansion. Gene structural analysis revealed that 64 *JmAP2/ERF* genes contained introns. Gene evolution analysis among Juglandaceae revealed that *J. mandshurica* is separated by 14.23 and 15 Mya from *Juglans regia* and *Carya cathayensis*, respectively. Based on promoter analysis in *J. mandshurica*, many cis-acting elements were discovered that are related to light, hormones, tissues, and stress response processes. Proteins that may contribute to cold resistance were selected for further analysis and were used to construct a cold regulatory network based on GO annotation and JmAP2/ERF protein interaction network analysis. Expression profiling using qRT-PCR showed that 14 *JmAP2/ERF* genes were involved in cold resistance, and that seven and five genes were significantly upregulated under cold stress in female flower buds and phloem tissues, respectively. This study provides new light on the role of the *JmAP2/ERF* gene in cold stress response, paving the way for further functional validation of *JmAP2/ERF* TFs and their application in the genetic improvement of *Juglans* and other tree species.

## 1. Introduction

Transcription factors (TFs) are the main class of regulatory proteins and play key roles in various abiotic and biotic stress responses in plants [1]. Among these TFs, the APETALA2/ethylene responsive element-binding factor (AP2/ERF) superfamily is one of the largest transcription factor families in plants, playing significant roles in plant growth and development, hormonal regulation, biotic and abiotic stress responses [2,3]. Members of this family contain one or two highly conserved AP2 domains, which is composed of 60–70 amino acid residues with typical helix-turn-helix structures [4,5]. The AP2 domain can bind the cis-acting elements, such as GCC box (AGCCGCC), dehydration-responsive element/C-repeat element (DRE/CRT, A/GCCGAC), to regulate transcription or gene expression [5,6]. Based on the number of AP2 domains and the sequence similarities, the AP2/ERF superfamily genes are categorized as AP2 (APETALA2), DREB (dehydration-responsive-element-binding), Soloist (few unclassified factors), RAV (related to ABI3/VP), and ERF (ethylene-responsive-element-binding) [5,7].

The AP2/ERF superfamily TFs have been identified in diverse plant species, including *Arabidopsis thaliana* [5], wheat (*Triticum turgidum*) [8], peanut (*Arachis hypogaea* L.) [9], tomato (*Lycopersicon esculentum* Miller) [10], common andrographis (*Andrographis paniculate*) [11], dark jute (*Corchorus olitorius* L.) [12], and Chinese cherry (*Prunus pseudocerasus*) [13]. The AP2 subfamily binds to the GCAC(A/G)N(A/T)TCCC(A/G)ANG(C/T) element and has an essentially important impact on reproductive organ development, including the flower, ovule, spikelet, seed, floral organ identity, and meristem maintenance [2,14]. This subfamily’s members can be classified into the ANT (one AP2 domain) and AP2 (two AP2 domains) groups [15]. The RAV subfamily binds to CAACA and CACCTG motifs, each member contains one AP2 domain and an additional B3 domain, involved in plant development, hormone signaling, and stress responses [16]. Conversely, the ERF and DREB subfamily members contain only a single AP2 domain, they can be distinguished based on the difference in the 14th and 19th amino acid residues in the AP2 domain [16,17]. The 14th and 19th amino acids of the ERF subfamily are alanine (Ala) and aspartate (Asp), respectively, which can specifically bind to the GCC-box element and are involved in the ethylene response, abiotic stress, and plant development [6,18]. Overexpression of *OsERF71* in rice elevated plant drought resistance [19]. The 14th and 19th amino acids of the DREB subfamily are valine (Val) and glutamate (Elu), respectively, which can specifically bind DRE/CRT elements. This subfamily performs significant roles in abscisic acid regulation and coping with cold and drought stress [20]. Among the DREB subfamily members, the C-repeat binding factor (CBF)/dehydration-responsive element binding (DREB1) is part of monophyletic group III (A1 group) in the DREB subfamily, playing vital roles in cold acclimation and freezing tolerance [21,22]. Overexpression of sweet pepper *CfCBF3* in tobacco triggers orthologs of *CBF3*-targeted genes and modifies fatty acid unsaturation, improving cold tolerance [23]. Additionally, other members also with an AP2 domain and different gene structure are regularly classified as the Soloist subfamily [5,12].

*Juglans mandshurica* is a deciduous tree belongs to the genus *Juglans* (Juglandaceae) [24]. The species is widely distributed throughout cold regions of China, South Korea, Japan, and Russia [25]. *J. mandshurica* is a good timber tree species due to of its hard and dense material and good elasticity [26]. The rich nutrition and high oil content in the fruit kernel of *J. mandshurica* can be used to make oil and health food. Juglone in fresh root bark, branch bark and green fruit peel has strong antibacterial and antitumor effects and is used in the field of medicine [27,28]. Furthermore, this species is an important resource for freeze-tolerant germplasm for genetic improvement, which can withstand freezing temperatures of −40 °C [26]. Most *Juglans* are weak in terms of cold resistance, so breeding varieties with strong cold resistance is an effective way to avoid or reduce freezing injury. However, due to the long breeding cycle and incompatibility of hybrids, research on the cultivation of new plant varieties using traditional breeding methods is limited [29]. Modern genetic engineering methods, such as genetic modification and gene editing (TALEN and CRISPR/Cas9), have great potential for molecular improvement in short periods and high efficiency, but that requires the knowledge of the specific role of gene families [30,31]. Hence, it is particularly important to identify gene families in plants that provide valuable information for genetic engineering breeding. Many studies have shown that the AP2/ERF TFs are significant regulators involved in plant growth and development, hormone regulation and stress response. *J. mandshurica* is considered an important germplasm resource for genetic improvement of stress resistance, but no systematic study has been performed on the *AP2/ERF* superfamily genes in *J. mandshurica*.

Here, we comprehensively conducted genome-wide identification and characterization of the AP2/ERF superfamily in *J. mandshurica* and analyzed the expression pattern to identify crucial cold responsive genes at different cold stress (i.e., 4 °C, −10 °C, −20 °C, −30 °C and −40 °C). The aims of this study included: (a) obtaining complete information on all members of the AP2/ERF superfamily for *J. mandshurica*; (b) investigating the phylogenetic and evolutionary relationship of *JmAP2/ERF* genes; (c) demonstrating the expression pattern and functional characteristics of *JmAP2/ERF* genes under cold stress; and (d) providing valuable resources for genetic engineering breeding in plants.

## 2. Results

### 2.1. Identification and Phylogenetic Analysis of JmAP2/ERF Genes

The HMMsearch programmer with the HMM profile (PF00847) was performed in the *J. mandshurica* genome; 192 putative members were detected. A total of 184 members were characterized as candidate *JmAP2/ERF* genes by NCBI Batch CD verification and filtering. The *JmAP2/ERF* superfamily was divided into five subfamilies, *JmAP2*, *JmRAV*, *JmSoloist*, *JmDREB* and *JmERF*, based on the sequence features, numbers of AP2 domains and the existence of the B3 domain (Table 1).

Each subfamily member was named and distinguished according to their gene annotation and taxonomic information in this study (Table S1). Twenty-seven genes were classified into the *JmAP2* subfamily, containing twenty-four genes with two consecutive AP2 domains and three genes (*JmAP2-02*, *JmAP2-09*, *JmAP2-10*) with one AP2 domain. The *JmRAV* subfamily had six genes, each with a C-terminal AP2 and B3 domain. In addition, the *JmSoloist* subfamily had four members. The remaining 147 genes with one AP2 domain were all located in either the *JmDREB* or *JmERF* subfamilies, had 57 and 90 members, respectively. Then, the physical properties of *JmAP2/ERF* superfamily genes were analyzed, including isoelectric point (pI), molecular weight (MW), CDS length and subcellular localization (Table S1). The MWs of the predicted proteins varied from 9.77 kDa (JmERF-04) to 86.06 kDa (JmAP2-01), and the pI values were 4.62 (JmERF-75) to 10.11 (JmERF-37). The outcomes of predicted subcellular localization revealed that 176 JmAP2/ERF members were localized in the nucleus (Table S1).

**Figure 1:**
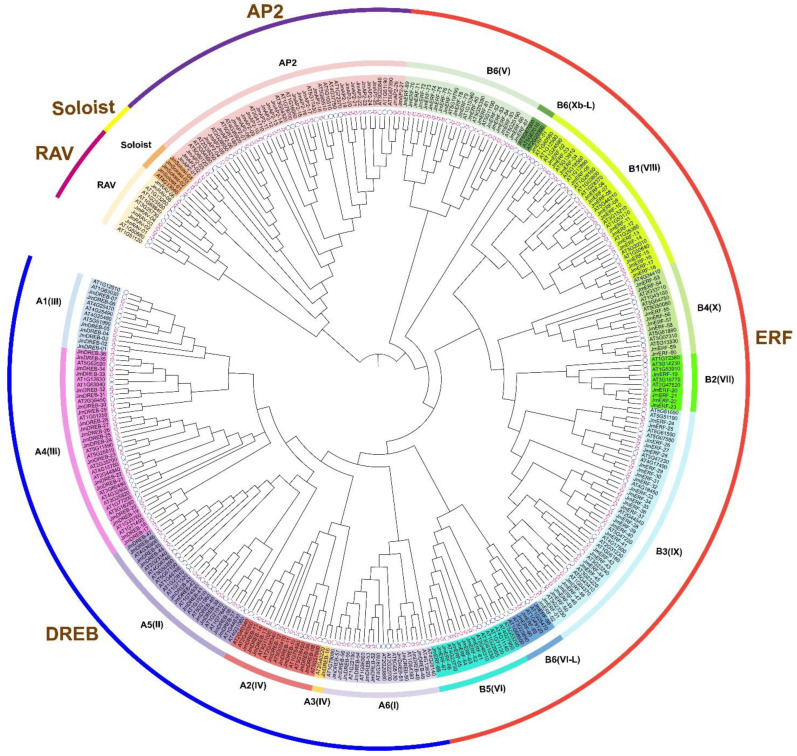
Phylogenetic analysis of AP2/ERF TFs in *J. mandshurica* and *A. thaliana*. Each subfamily and group were shown in various colors. Markers of individual genes: red stars represent *JmAP2/ERF*, and blue circles represent *AtAP2/ERF*.

To examine the evolution of the *JmAP2/ERF* genes, an ML phylogenetic evolutionary tree was constructed using amino acid sequences. The 184 *JmAP2/ERF* genes were grouped with the 147 *AtAP2/ERF* genes, and the *AP2/ERFs* could be split into five subfamilies (*AP2*, *RAV*, *Soloist*, *DREB*, *ERF*) with 10 groups (*DREB*-I to IV and *ERF*-V to X) (Table 1 and Figure 1). The current phylogenetic tree results indicated that classification of *AP2/ERF* superfamily genes in *J. mandshurica* were similar to *Arabidopsis* and peanut [9]. The presence and location of introns and the analysis of motifs outside the AP2/ERF structure also supported the reliability of this clustering. All genes in the five subfamilies exhibited clearly distinguished branching in the ML tree (Figure 1). Genes from the *RAV*, *AP2*, and *Soloist* subfamilies were clustered into a large clade, which was congruent with previous studies [12]. Moreover, Group III (*JmDREB*) and Group IX (*JmERF*) contained the most members at 27 and 29, respectively, yet there were no members of the Xb-L group in the *JmERF* subfamily. The number of *JmAP2* and *JmERF* subfamily genes was significantly increased compared with *Arabidopsis*, these changes contribute to coping with more complex biotic and abiotic stresses for *J. mandshurica*.

### 2.2. Gene Structure and Conserved Motif Analysis of JmAP2/ERFs

**Figure 2:**
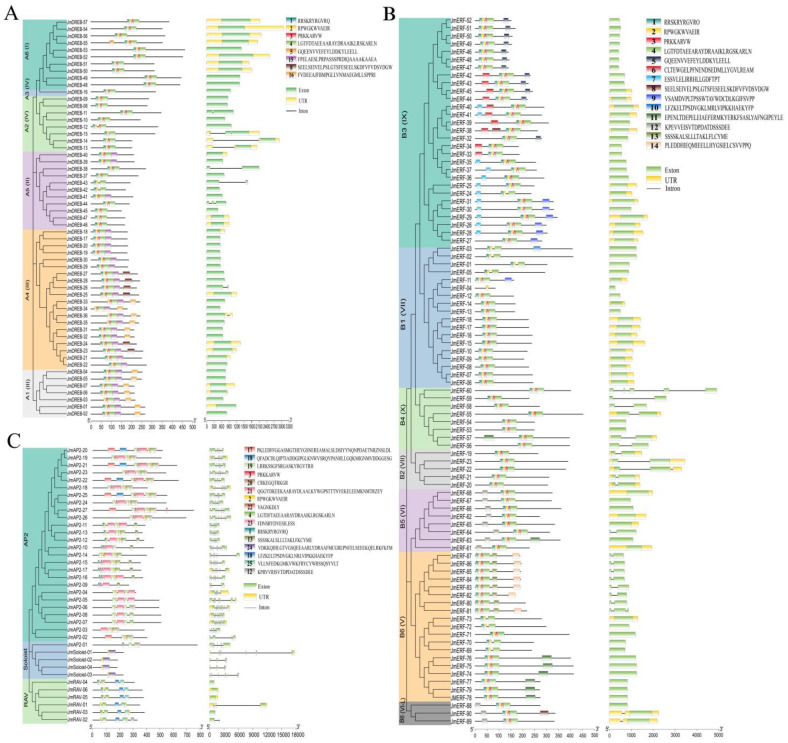
Conserved motifs and exon–intron structures of *JmAP2/ERF* genes. (**A**) *JmDREB* subfamily; (**B**) *JmERF* subfamily; (**C**) *JmAP2*, *JmSoloist* and *JmRAV* subfamilies. Different-colored boxes symbolize motifs.

Gene structure studies among AP2/ERF superfamily members help us understand the conserved features and evolutionary differences of this family’s proteins. Gene structure analysis revealed that the number of introns varied among the *JmAP2/ERF* family genes. The *JmAP2* subfamily genes contained between 3 to 10 exons and 2 to 9 introns, indicating significant variability. Members of the *JmSoloist* subfamily contained 3 to 6 introns, while only two members of the *JmRAV* subfamily contained introns (*JmRAV-01*, *JmRAV-02*). Among 147 *JmDREB* and *JmERF* members, most had zero or one intron; only *JmDREB44*, *JmERF22* and *JmERF81* contained two introns, while *JmERF60* contained four introns. Most introns were present in the A2, B2, B4 and B6 groups of *JmDREB* and *JmERF* subfamilies. The results of the five subfamilies revealed that some genes contained exons and introns without UTR regions, indicating these genes have a specific evolutionary process (Figure 2; Table S1).

To further investigate the classification and function of JmAP2/ERF encoding proteins in different groups, 25 conserved motifs were analyzed using the MEME tool, and some were present in specific subfamilies or groups (Figure 2). Six conserved motifs were identified in JmDREB subfamily protein sequences, with motifs 1, 2, 3, and 4 found in all AP2 domain regions. The members within a group contained a similar number and type of motif, whereas they were different across groups. For example, motif 16 was present within the seven members of the A1 group and JmDREB31-36 of the A4, while JmDREB23-27 of A4 all contained motif 8 (Figure 2A). The distribution of motifs 1-4 in the JmERF subfamily was similar to that of the JmDREB subfamily, which contained 16 motifs. Motifs 5, 6, 7, and 9 were present in the B3 group, motif 12 was found only in the B5 and B6 (VI-L) groups, and motifs 11, 13, and 15 were present in the B6 (V) group (Figure 2B). Sixteen motifs were found in the JmAP2, JmRAV and JmSoloist subfamilies and were distributed as 37 protein sequences (Figure 2C). Among them, motifs 2, 3, 4, 19, 21, and 23 existed in the AP2 domain of JmAP2 in a specific pattern. Motifs 10 and 25 were only located in the B3 domain of JmRAV, and motifs 2 and 24 were exclusively present in JmSoloist. Combined with intron–exon structure and conserved motif analysis, it was clear that members of the same group have similar characteristics, suggesting that most *JmAP2/ERF* genes are highly conserved in the evolution.

### 2.3. Chromosome Distribution, Duplication Events and Synteny Analysis of JmAP2/ERFs

**Figure 3:**
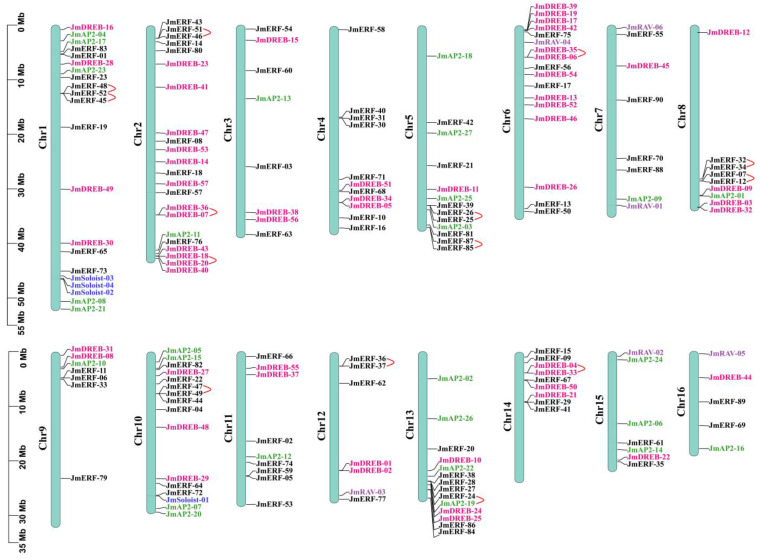
*JmAP2/ERF* gene distribution in *J. mandshurica* chromosomes. Subfamily genes are distinguished by different front hues. Pink gene ID: *JmDREB*; black gene ID: *JmERF*; green gene ID: *JmAP2*; blue gene ID: *JmSoloist*; purple gene ID: *JmRAV*. Tandem duplication is shown by red curved lines.

Based on the *J. mandshurica* genome information, 183 genes from 184 identified *JmAP2/ERF* genes were mapped to 16 chromosomes except for *JmERF-78* (Figure 3 and Table S2). The distribution of these genes across the chromosomes varied widely. The *JmAP2/ERF* genes density per chromosome ranged from 2.7% to 12.0%. The number of *AP2/ERF* genes was greatest on chromosome 2 (22), chromosome 16 had the fewest (5), and the number on the other chromosomes ranged from 7 to 21. Most of the genes were on both ends of the chromosomes, which was similar to studies in *Arabidopsis* and other species [5,32,33]. The distribution similarity of genes on chromosomes indicates functional consistency.

**Figure 4:**
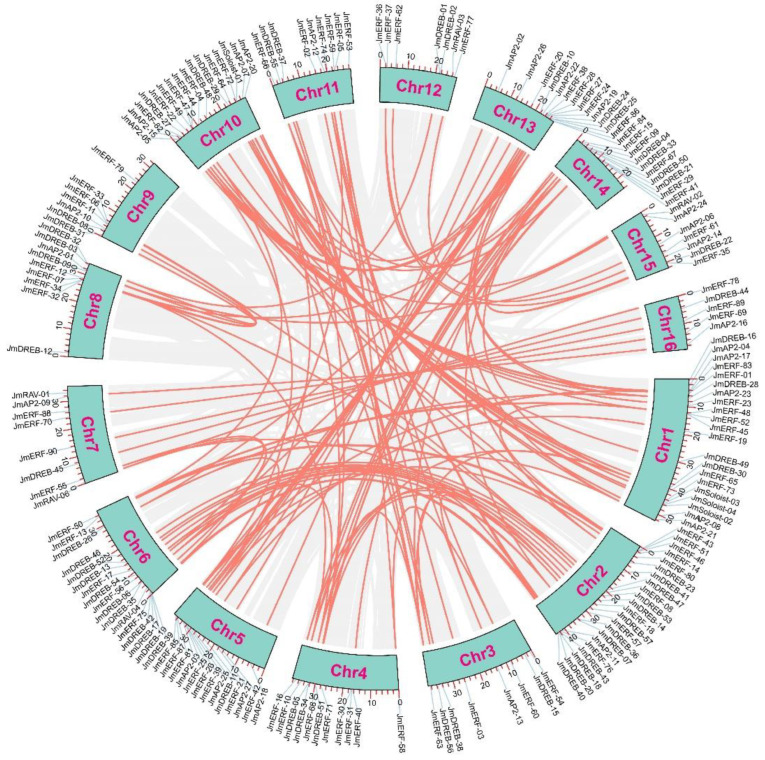
Circos schematic of segmental duplications of *JmAP2/ERFs*. Gray and orange lines indicate all syntenic blocks and segmented *duplicated* gene pairs in the *J. mandshurica* genome, respectively.

**Figure 5:**
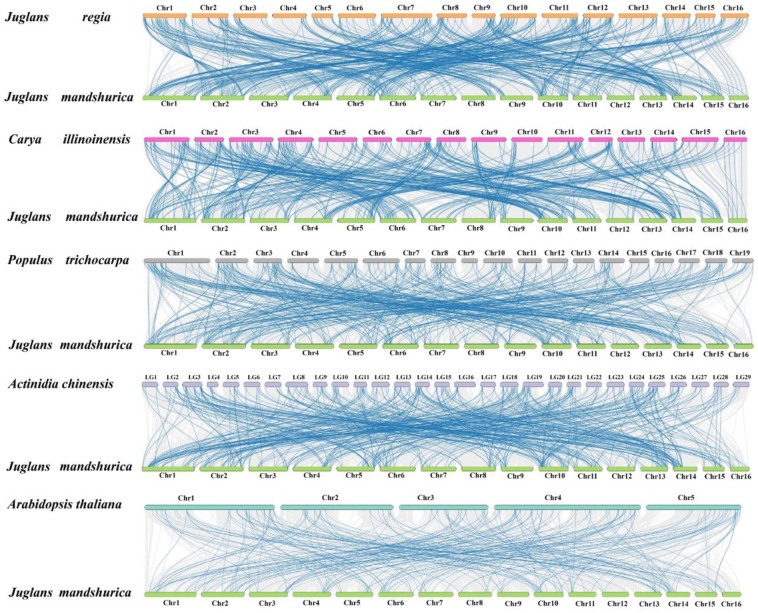
Synteny analysis of *AP2/ERFs* between *J. mandshurica* and *J. regia*, *C. illinoinensis*, *P. trichocarpa*, *A. chinensis*, *A. thaliana*. Gray lines represent collinear relationship within *J. mandshurica* and five species, blue lines represent syntenic *AP2/ERFs* pairs.

Gene duplication events (segmental and tandem duplication) are the driving force behind the evolution of gene families in plants. To elucidate the evolution mechanism of the *JmAP2/ERF* superfamily, large-scale gene replication events were investigated in *J. mandshurica* and other species. In total, 136 duplicated gene pairs were detected, including 14 tandem and 122 segmental gene duplications in *J. mandshurica* (Figure 3 and Figure 4). This result implied that segmental replication events play a more important role, when the JmAP2/ERF family copes with various environmental changes than tandem replication. Similar discoveries have been found in *Arabidopsis* [5], peanut [9], tomato [10], and dark jute [12]. To evaluate the evolutionary pattern of the *JmAP2/ERF* superfamily, comparative orthologous analysis was performed among *Juglans regia*, *Carya illinoinensis*, *Populus trichocarpa*, *Actinidia chinensis* and *A. thaliana* (Figure 4). In total, 433, 408, 392, 404, and 205 orthologous gene pairs were found between *J. mandshurica*, *J. regia*, *C. illinoinensis*, *P. trichocarpa*, *A. chinensis*, and *A. thaliana*, respectively (Figure 5 and Tables S4–S9). Moreover, the Ka/Ks value for tandem duplication was 0.11 to 0.29, with an average of 0.14, while Ka/Ks for segmental duplication was 0.05 to 0.46, with an average of 0.21. These segmental and tandem duplications occurred ~22.50–70.96 Mya, respectively. The Ka/Ks ratios of orthologous gene pairs between *J. mandshurica* and *J. regia* (0.24), *C. illinoinensis* (0.24), *P. trichocarpa* (0.16), *A. chinensis* (0.18), and *A. thaliana* (0.15) were strongly subjected to pure selection. The divergence times were 14.23, 15.00, 53.14, 55.20, and 78.38 Mya, respectively (Tables S3–S9).

### 2.4. Analysis of Cis-Acting Elements in JmAP2/ERF Promoters

**Figure 6:**
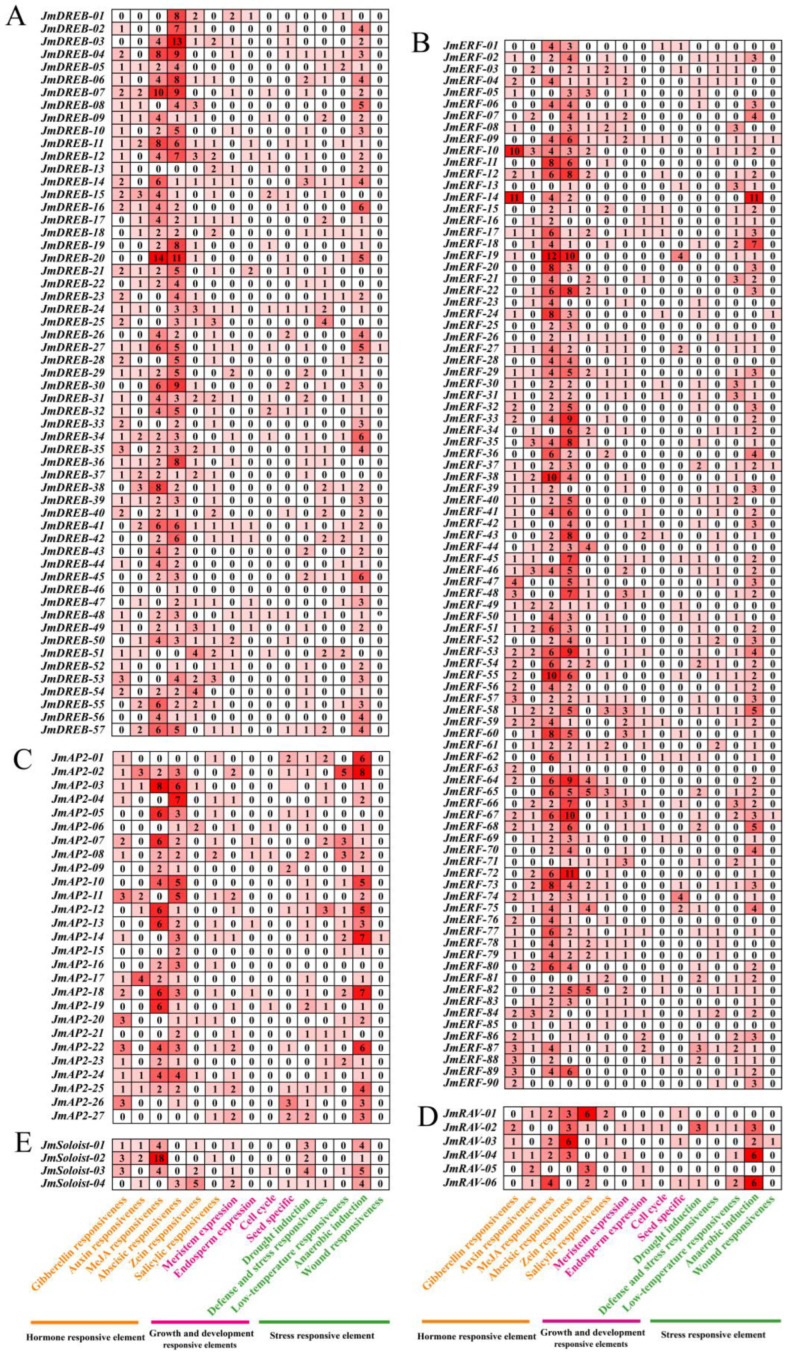
Cis-acting elements analysis of *JmAP2/ERFs* promoters. (**A**) *JmDREB* subfamily; (**B**) *JmERF* subfamily; (**C**) *JmAP2* subfamily; (**D**) *JmRAV* subfamily; (**E**) *JmSoloist* subfamily. The shadows and black numbers represent the number of cis-acting elements in three different biological processes.

Cis-acting elements can be recognized by TFs and participate in tissue specific and stress response gene expression. To further investigate the roles and regulatory mechanisms of *JmAP2/ERFs* in various biological processes, particularly in response to cold stress, the sequences 2000 bp upstream of the *JmAP2/ERFs* were identified using PLANTCARE. In total, 49 types of cis-acting elements were detected, and 11 types of light-responsive elements were the most abundant (Table S9). Subsequently, the cis-acting elements related to the growth and development response, hormone response and stress response in the *JmAP2/ERF* promoter were further analyzed (Figure 6).

In general, the *JmDREB* subfamily genes are more likely to regulate stress responses, while the *JmERF* subfamily genes are involved in plant hormone regulation, which is related to the homeopathic elements they contain (Figure 6A,B). Among the identified cis-acting elements, those related to growth and development response were abundant (10 types), such as cat-box, circadian control, AACA and GCN4 motifs. These elements are involved in meristem development, circadian control, and the endosperm growth response. Ten types of hormone responsive elements were identified, including those associated with abscisic acid (ABRE), auxin (TGA-element, TGA-box, AuxRR-core), MeJA (TGACG-motif, CGTCA-motif), gibberellin (P-box, TATC-box, GARE-motif) and salicylic acid (TCA-element, SARE). Elements related to low temperature responsiveness (LTR) and drought induction (MBS), except for anaerobic induction elements were also identified in many *JmAP2/ERF* promoters. Analysis of the stress response elements revealed that 92, 77 and 64 genes contained drought-induction, low-temperature response, defense, and stress response elements (TC-rich repeats), respectively. Twenty-six genes contained both low-temperature response (LTR and/or DRE) elements, which was more likely to regulate the stress response (Table S10). The variety and functions of these cis-acting elements provide insight into the biological functions of *JmAP2/ERF* genes.

### 2.5. Interaction Network Analysis of JmAP2/ERF Proteins

**Figure 7:**
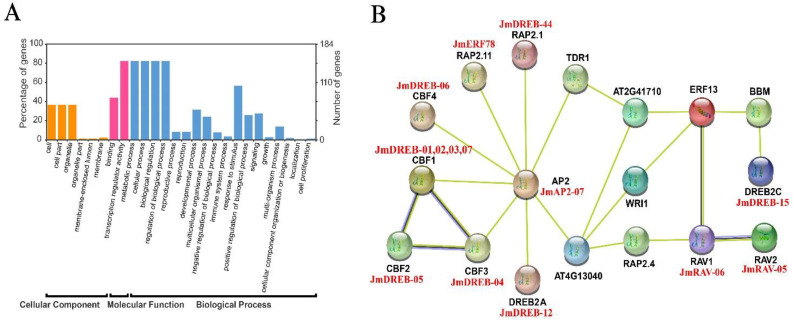
GO annotation analyses and interaction network of JmAP2/ERF proteins. (**A**) GO annotation analyses of the common JmAP2/ERF proteins expressed. (**B**) Protein interaction network of representative JmAP2/ERF proteins based on the orthologs in *Arabidopsis*.

The GO annotation analysis of all 184 JmAP2/ERF superfamily proteins revealed 18 biological processes, among which roles in metabolism, cellular processes and the regulation of biological processes were predominant (Figure 7A and Table S11). In these biological processes, 104 JmAP2/ERF proteins were involved in the response to stimulus processing, consistent with the function of AP2/ERF proteins. The terminology labels of these *JmAP2/ERF* genes could help us understand the protein’s functions. To further study the potential function and synergy of JmAP2/ERF TFs in regulation, a protein interaction network was constructed using *Arabidopsis* orthologous proteins (Figure S1). The results showed that the five proteins: JmAP2-07, JmAP2-14, JmSoloisy-01, JmRAV-06 and JmERF-35, serve as crucial nodes that interact with other TFs to form a complicated regulatory network. Some representative proteins were selected to further analyze the cold regulation network (Figure 7B). This network includes four proteins similar to CBF1 (JmDREB-01, JmDREB-02, JmDREB-03, and JmDREB-07), one that is similar to CBF2 (JmDREB-05), and one that is similar to CBF3 (JmDREB-04). These proteins were involved in more powerful cold-response networks, because CBF1, CBF2, and CBF3 were the key TFs in the core mechanism of cold stress mediated by the ICE1-CBF-COR signaling pathway. Similarly, JmDREB-44, JmERF-78, JmRAV-05, and JmRAV-06 protein sequences showed high homology with RAP2.1, RAP2.11, RAV-02, and RAV-01 that were involved in the response to low-temperature interaction network. Additional proteins that might be involved in the response to low-temperature include JmDREB-44, JmERF-78, JmRAV-05, and JmRAV-06, which corresponds to RAP2.11, RAV-02, and RAV-01, respectively. Furthermore, CBF4 was involved in the response to drought stress but not to low temperatures, JmDREB-06 and CBF4 might have comparable functions. Based on the protein sequences of JmDREB-12 and JmDREB-15, which have high homology with DREB2A and DREB2C, respectively, this indicates that they were involved in the responses to low-temperature and drought stress.

### 2.6. Expression Profiles of JmAP2/ERF Genes under Cold Stress Using qRT-PCR

**Figure 8:**
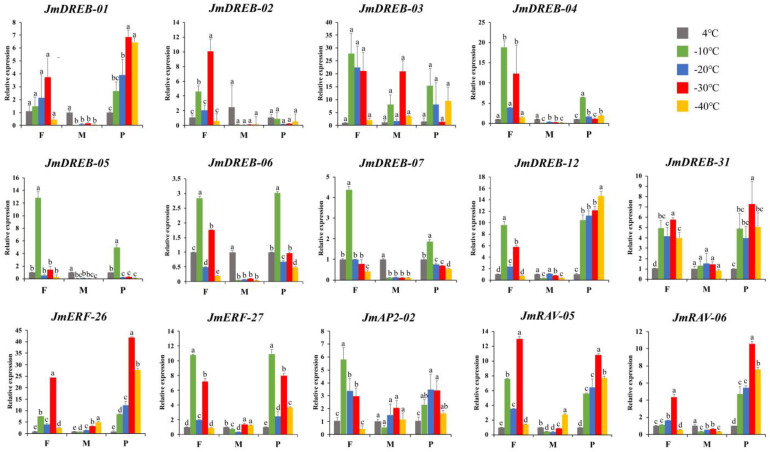
Expression profiles of fourteen *JmAP2/ERF* genes in response to cold stress treatments in different tissues. The female flower buds (F), male flower buds (M) and phloem tissues (P) were sampled at 4 °C, −10 °C, −20 °C, −30 °C, and −40 °C after 10 h of cold treatment. The data shown are the mean values ± SDs of three replicates. Means denoted by the same letter do not significantly differ at *p* ≤ 0.05 as determined by the SNK test.

Based on the results of the phylogenetic, cis-acting element, GO annotation and PPI network analyses, 14 genes related to the cold stress response were screened. To further understand the molecular mechanism of cold tolerance in *J. mandshurica*, these *JmAP2/ERF* genes were used to verify their function and expression by qRT-PCR in three tissues (female flower buds, male flower buds and phloem tissues). The results demonstrated that the 14 selected *JmAP2/ERF* genes were differentially expressed in the three tissues under different cold stress conditions, indicating the regulatory functions of these genes were tissue specific (Figure 8). There were seven genes that had relative expression levels higher than 10 in the female flower buds (*JmDREB-02*, *JmDREB-03*, *JmDREB-04*, *JmDREB-05*, *JmERF-26*, *JmERF-27*, *JmRAV-05*) and five genes where the relative expression levels were higher than 10 in phloem tissue (*JmDREB-03*, *JmDREB-12*, *JmERF-26*, *JmERF-27*, *JmRAV-05*). Three genes (*JmDREB-03*, *JmERF-26*, and *JmERF-27*) were highly expressed in phloem tissue buds and female flower buds. However, there were fewer genes with higher relative expression in male flower buds than in female flower buds and phloem tissues, which can be the reason that the male flower buds’ sap flow stopped, prematurely entering dormancy. Moreover, two main expression patterns of these genes at different low temperatures were presented: one was highly expressed at −10 °C, and the other was highly expressed at −30 °C. The genes *JmDREB-04*, *JmDREB-05*, *JmDREB-06* and *JmDREB-07* were revealed as low-temperature sensitive genes that were highly expressed at −10 °C in response to cold stress. Similarly, expression levels of *JmDREB-01*, *JmDREB-02*, *JmDREB-12*, *JmERF-26*, *JmRAV-05*, and *JmRAV-06* gradually increased with decreasing temperature, reaching maximum values at −30 °C, suggesting that these genes play a key function when cold stress is at a greater intensity. These results indicate that *JmAP2/ERFs* cooperate to resist the damage caused by cold stress.

## 3. Discussion

The *AP2/ERF* superfamily genes, specific to plants, regulates growth, development, and abiotic stress responses [2]. They are vital candidate genes for improving growth and development, and abiotic stress in plants, responding to variable stresses, such as flower development (*PhAp2A*, *PhAp2B*, *PhAp2C*) [2], drought (*AtCBF4*, *OsERF71*) [19], low-temperature (*AtCBF1*, *AtCBF2*, *AtCBF3*, *CfCBF3*) [2,23], and heat (*CmDREB6*, *ZmDREB2A*) [34]. Therefore, a genome-wide investigation of *AP2/ERF* gene families will help elucidate plant growth roles, molecular processes, and environmental adaptation. However, the *AP2/ERF* superfamily gene information is limited because whole genome studies have not been performed in *J. mandshurica*. In this paper, the AP2/ERF superfamily of *J. mandshurica* was identified and analyzed for the first time using extensive bioinformatics methods, including phylogenetic, gene structure, motif distribution, chromosome localization, and interaction network analyses, as well as evolutionary relationships.

### 3.1. Identification of the AP2/ERF Superfamily

With the studies of TFs having evolved into an integral element in functional genomics research, the genome-wide identification and functional analysis of TFs in plants becomes particularly significant [2,3]. Here, we identified 184 AP2/ERF superfamily TFs in the *J. mandshurica* genome. All putative *JmAP2/ERF* genes were divided into five subfamilies: *JmAP2*, *JmRAV*, *JmSoloist*, *JmERF* and *JmDREB*, and each subfamily contained 27, 6, 4, 57, and 90 members, respectively. The number of genes within each subfamily varied significantly in these plants, but they all followed a similar pattern, with the largest number belonging to the ERF subfamily, followed by DREB, RAV, or Soloist. This indicated that AP2/ERF TFs may have a common progenitor before separation (Table S12) [9,12]. Among these subfamilies, significant differences in ERF and DREB gene counts demonstrated that these genes increased throughout evolution and play key roles in plant growth and development, which is consistent with a previous study [13,18]. The quantity and structural characteristics of transcription factors in the gene family are related to the size of the genome and the effects of the long-term evolution of plant species [35]. In contrast to *A. thaliana* (147), *Nelumbo nucifera* (121), *C. olitorius* (119) and *P. pseudocerasus* (67), the members of the AP2/ERF family of *J. mandshurica* were present at higher levels but were less than that in *Brassica napus* L. (531), *Helianthus annuus* L (288), *Triticum turgidum* ssp. *Durum* (271), *A. chinensis* (268) and *C. illinoinensis* (202) (Table S12). Differences in the number of *AP2/ERFs* among the plants can be explained by genetic evolution and duplication. Differences in the molecular weight (9.77–86.06 kDa) and isoelectric point (4.62–10.11) of *JmAP2/ERF* indicate putative differences in *J. mandshurica* [36]. A predicted subcellular localization showed that JmAP2/ERF TFs are primarily localized to the nucleus and can function in transcription and post-transcriptional modification; similar results have been found in other plant studies [37].

### 3.2. The Characteristics Information of AP2/ERF Superfamily in J. mandshurica

Conventionally, the conserved motifs in TFs are related to protein interactions, transcriptional activity, and DNA binding. The number and location of introns in gene structure analysis also provides clues to the evolutionary relationships among proteins [5]. In this study, the conserved motifs and intron–exon distribution of *JmAP2/ERFs* were analyzed to obtain a better understanding of the structural features of *JmAP2/ERFs.* Analyzing the exon-intron structure of the *JmAP2/ERF* gene revealed that 64 members of this family possessed introns, including 27 *JmAP2* genes, 2 *JmRAV* genes, 4 *JmSoloist* genes, 9 *JmDREB* genes, and 22 *JmERF* genes. Among them, the *JmAP2* subfamily contained more introns than other families; however, zero or few introns were observed in the *JmERF* and *JmDREB* subfamilies; similar results have been found in *C. olitorius* L. [12], *A. hypogaea* L. [32], and *C. illinoinensis* [33]. This seemed to be related to the 122 pairs of segmental duplication events in this study and further confirmed that segmental duplication leads to intron loss with a higher probability than gain [36]. The high variation in *JmAP2/ERF* exon–intron structure may indicate that the *J. mandshurica* genome has undergone a large differentiation (such as tandem and segmental events) during the process of evolution, resulting in the functional differentiation and structural diversity of the *JmAP2/ERF* gene family [35]. Identifying and distributing conserved motifs may help predict structural variation, functional differences, and gene evolution across family members [16,29]. The conservative motif analysis identified motifs 2 and 4 in all JmAP2/ERF protein sequences, which were related to the AP2 domain. The AP2 subfamily protein sequences have motifs 17, 18, and 23, whereas Soloist and RAV have motifs 24 and 25, indicating that different subfamilies of genes are highly conserved and play an important role in their subfamilies. Similar results have been found in *Dimocarpus longan* Lour [38], and *Fagopyum Tataricum* [39], indicating that a high degree of evolutionary conservation is an important basis for subfamily classification. These findings show that different preserved motifs were arranged, but most genes in the same subfamily contained similar conserved motifs and gene structures, indicating that they may possess similar structures and biological functions.

Cis-regulatory elements (CREs) play a key role in the *JmAP2/ERF* gene expression, which may control other genes under conditions of stress to establish complex regulatory networks [6,18], such as cold stress. In the CREs analysis of the *JmAP2/ERF* genes, 27 genes contained low-temperature responsiveness elements (LTRs), and 3 genes contained dehydration responsive elements (DREs). In the study of cherry flower bud, these CREs were also identified and proved to play an important role in the process of low temperature stress response [13]. Members of the III (A1) group in the *AP2/ERF* superfamily belong to the *CBFs*, which are key transcription factors in cold acclimation and response [40]. Transgenic overexpression of the barley *HvCBF4* and Arabidopsis *AtCBF1* genes in rice and tomato increases resistance to low temperature stress, and the maximum photochemical efficiency of photosystem II (F-v/F-m) in transgenic plants was higher than that in nontransgenic plants [41,42]. Moreover, phylogenetic analysis indicated that *JmRAV-05* and *JmRAV-06* were closely related to *At1G13260* (*AtRAV1*), which was upregulated in response to low temperature [43], *JmRAV-05* and *JmRAV-06* may also have similar expression patterns and functions.

### 3.3. Gene Duplication Events Are Vital Factors in the JmAP2/ERF Superfamily Evolution

Gene duplication (tandem and segmental duplication) plays a key role in plant adaptive evolution [35]. Generally, tandem duplication produces gene clusters or hot regions, while segmental duplication generates homologous genes, which combine to result in the evolution of the gene family [36,44]. Chromosomal mapping revealed an uneven distribution of *JmAP2/ERFs* on 16 *Chrs*, and there were gene clusters or hot regions on chr02, chr06 and chr13. Significantly, *J. mandshurica* chromosomes have 122 segmental and 14 tandem duplications, while 89.7% of gene duplication events were segmental, indicating that segmental duplications play a key role in the evolution of the *JmAP2/ERF* gene family. The paralogous gene pair numbers of the *AP2/ERF* subfamily vary among plants: for example, there are 165 duplication pairs in *C. illinoinensis*, 95 in *Arachis hypogaea* L, 90 in *H. annuus* L, 51 in *A. thaliana*, and 11 in *C. olitorius* L, all of which were lower than those in *J. mandshurica*. Thus, this variation in the number of *AP2/ERF* genes in plants could be due to different gene duplication events. In addition to tandem and segmental duplications, whole-genome duplication (WGD) events also affect the number of family members [12] A previous study of the chromosome-level genome assembly in *J. mandshurica* revealed that *J. mandshurica* and *J. regia* originated from a common ancestor, with both species undergoing two whole-genome duplication events [31]. Therefore, tandem, segmental, and whole-genome duplication events might be responsible for the evolution of the *AP2/ERF* gene family. Microsynteny analysis of the *AP2/ERF* genes throughout the *J. mandshurica* family revealed that *J. mandshurica* is significantly associated with *AP2/ERF* genes in *J. regia* and *C. illinoinensis*, providing vital information on their evolution (Figure 5). The mean Ka/Ks ratio of ortholog gene pairs of *J. mandshurica* with *J. regia* (0.24) and *C. illinoinensis* (0.24), implies that the evolution of *JmAP2/ERF* genes was strongly subject to pure selection (Tables S5 and S6). This may be related to the strong environmental adaptability of *J. mandshurica* in the evolutionary process.

### 3.4. Exploring AP2/ERF Genes Function and Expression Profile in J. mandshurica

Identification and functional verification of TFs are the basis for plant genetic improvement, which can provide important candidate gene resources. Previous reports have shown that AP2/ERF TFs can be potential candidates for plant improvement because they are key regulators in different plant development processes and various stress responses [12]. In *J. mandshurica*, female flower buds, male flower buds, and phloem tissues are the main overwintering organs with strong cold resistance [26]. In this study, we used these tissues to perform cold stress treatment with different gradients. Based on the results of cold acclimation and cold stress in previous studies [45,46], we selected a 10 h treatment time-point used for the cold stress treatment of current-year branches from *J. mandshurica*. Then, qRT-PCR was used to study the function and expression profile of the *JmAP2/ERF* genes in response to cold stress. This analysis identified seven genes in the female flower buds, five genes in the phloem and one in the male flower buds that were highly expressed, suggesting that these are sensitive to cold stress to different degrees, and the physiological states of these tissues are strongly related to cold resistance, consistent with previous studies [47]. The functions of most AP2/ERF genes have been well characterized in *Arabidopsis* [2]. The expression profiles of 14 *JmAP2/ERF* genes also displayed a strong correlation with cold stress (Figure 8). Among these members, genes homologous to *AtCBFs*, which include *JmDREB-01*, *JmDREB-02*, *JmDREB-03*, *JmDREB-07* (*AtCBF1*), *JmDREB-05* (*CBF2*) and *JmDREB-04* (*CBF3*), may be essential genes in cold stress and resistance. In transgenic rice and *Arabidopsis*, overexpression of the *CBF1/DREB1B*, *CBF2/DREB1C*, and *CBF3/DREB1* genes increased low-temperature tolerance [48,49]. *JmERF-27* was significantly increased at −10 °C, which was homologous to the *At5G47230*, as a cold-upregulated gene [50]. Similarly, *JmERF-26* was significantly upregulated at −30°C in phloem tissue; it was homologous to the *At4G17490* that is involved in phloem histogenesis and the response to cold stress [51]. Not only that, *CBFs* can directly regulate the expression of *COR* genes, such as the function of *JmDREB-44* that is homologous to *RAP2.1*. Comparing the analysis results of the cis-acting elements and qRT-PCR, *JmCBF* genes are rapidly induced by cold stress, and bind to the promoter regions of *JmCORs* to activate their transcription, thereby enhancing the freezing tolerance of *J. mandshurica* (Figure 6 and Figure 8). These results confirmed that these *JmAP2/ERF* genes play significant roles in the cold stress response.

Cold accumulation is a key factor affecting a plant’s response to low temperature to reduce or avoid stress damage [13]. However, knowledge regarding plant responses to low temperature is still quite basic. In this study, we treated three tissues at five temperatures (4 °C, −10 °C, −20 °C, −30 °C, −40 °C) for 10 h to simulate the process of cold accumulation. Interestingly, the *JmAP2/ERF* genes presented two high expression patterns at −10 °C and −30 °C, which could be related to the cold-sensing system of *J. mandshurica*. During the study of the dormancy phase transition in Chinese cherry flower buds, *PpcAP2/ERF* genes also present a similar expression profile. The high expression levels of most *PpcAP2/ERF* genes in flower buds occurred in two stages of low temperature accumulation at 4 °C [13]. These genes coordinated expression to better adapt to freezing stress by activating multiple pathways, such as Ca^2+^, hormones, and cold signals [52], but the transcriptional regulatory cascade of *AP2/ERFs* in the cold stress signaling pathway requires further research. The results of this study provide new insights into the potential functional roles of AP2/ERF superfamily members and will help to select candidate AP2/ERF genes for further functional research. To date, only the NAC [35] and WAK [53] family TFs have been identified in *J. mandshurica*, and their expression and function were studied in fruit development and cell wall signaling. However, the lack of established genetic transformation technology for the non-model *J. mandshurica* greatly limits the ability to verify gene functions through homologous expression studies. In future studies, mature almond genetic transformation for *J. mandshurica* should be established as soon as possible, to verify the function and role of TFs more accurately through homologous expression.

## 4. Materials and Methods

### 4.1. Identification and Sequence Analysis of JmAP2/ERF Genes

Genome-wide data of *J. mandshurica* were downloaded from NGDC (https://ngdc.cncb.ac.cn/, PRJCA006358 (accessed on 8 October 2021)) [54]. The Markov (HMM) profile of the AP2 domain (PF00847) was obtained from the Pfam database (https://pfam.xfam.org/ (accessed on 8 October 2021)). HMMER 3.0 was used to query the AP2/ERF proteins from the *J. mandshurica* genome database with a default E-value < 1 × 10^−5^. The Batch NCBI CD-Search Tools (https://www.ncbi.nlm.nih.gov/Structure/bwrpsb/bwrpsb.cgi (accessed on 9 October 2021)) were used for further validation of the retrieved gene sequences to confirm the predicted TFs in the AP2/ERF family (default parameter: E-value < 0.01, Maximum aligns is 500). The biochemical properties of AP2/ERF family proteins were determined using ExPASy ProtParam (https://web.expasy.org/protparam/) [55]. Subcellular localization of curated *AP2/ERF* superfamily gene products was predicted using WoLF PSORT (https://wolfpsort.hgc.jp/ (accessed on 16 October 2022)) [56].

### 4.2. Phylogenetic Analysis, Gene Structure Identification and Conserved Motif Distribution

The maximum likelihood (ML) phylogenetic tree was constructed by MEGA 7.0 using the Poisson model (1000 bootstraps) [57]. The Interactive Tree of Life (iTOL, https://itol.embl.de/ (accessed on 15 October 2022)) was used to process the graphical visualization of phylogenetic trees [58]. The structural characteristics of the *AP2/ERFs* were identified by TBtools using genomic files [59]. The conserved motifs of JmAR2/ERF proteins were analyzed using Multiple Em for Motif Elicitation (MEME, https://meme-suite.org/meme/tools/meme (accessed on 23 October 2022)) with the default parameter (where the maximum number of motifs is 25) [60]. Subsequently, the combined images were obtained through TBtools [59].

### 4.3. Chromosomal Distribution, Gene Duplications, and Evolutionary Analysis

The chromosomal distribution of JmAP2/*ERFs* was mapped using TBtools software according to the annotations of *J. mandshurica* genomic files. *AP2/ERF* duplication events were identified using the Multiple Collinearity Scan toolkit (MCScanX) of TBtools [59]. TBtools was used to calculate synonymous (Ks) and nonsynonymous (Ka) substitution values. Duplication (Mya, million years ago) and divergence times were estimated using the following formula: T = Ks/2λ (λ = 1.5 × 10^−8^) [29].

### 4.4. Cis-Acting Element Analysis

Sequences from approximately 2000 bp upstream of the *JmAP2/ERF* promoter were selected to identify potential cis-acting elements using the PlantCARE database (http://bioinformatics.psb.ugent.be/webtools/plantcare/html/ (accessed on 25 October 2022)) [61], and the results were visualized using TBtools [59].

### 4.5. Prediction of the Protein Interaction Network

Gene Ontology (GO) annotation of JmAP2/ERF protein sequences was analyzed using the eggNOG-mapper v2 tool (http://eggnog-mapper.embl.de/ (accessed on 27 October 2022)) [62]. Visualization analysis of GO annotation results was performed by applying WEGO v2.0 (https://wego.genomics.cn/ (accessed on 27 October 2022)) [63]. Protein–protein interaction (PPI) networks were constructed using STRING v11.5 (https://cn.string-db.org/ (accessed on 29 October 2022)) with a medium confidence (combined score >400). Representative JmAP2/ERF protein sequences were used as queries, and *A. thaliana* was used as a reference [64].

### 4.6. Plant Materials and Cold Stress Treatments

Plant materials of the current-year branches from *J. mandshurica* were collected from Northeast Forestry University, Harbin, Heilongjiang Province, China. Prior to being transported to the laboratory, the branches were cleaned with deionized water, and the incision was sealed with paraffin wax. These branches were divided into five groups and treated at a temperature of 4 °C, −10 °C, −20 °C, −30 °C or −40 °C for 10 h using a programmable temperature and humidity chamber (YG751-3, GUOLIANGYIQI). Then, three biological replicates of the plant tissues, including female flower buds (F), male flower buds (M), and phloem tissues (P) of different treatments were collected (Figure S2). All samples were immediately frozen in liquid nitrogen and stored at −80 °C until use.

### 4.7. RNA Extraction and qRT-PCR Analysis

Total RNA from three tissue samples was isolated using an RNA Extraction Kit (Tiangen Biotech, Beijing, China). The quantity and quality of all RNA samples were assessed by agarose gel electrophoresis and the A260/A280 ratio using a Nanodrop 2000 Spectrophotometer. The cDNA was prepared using the PrimeScript RT reagent kit with gDNA Eraser (TaKaRa, Kyoto, Japan). The qRT-PCR primers were designed using the online website Integrated DNA Technologies (https://sg.idtdna.com/pages (accessed on 20 November 2022)). A total of 14 putative cold-resistant *JmAP2/ERF* genes were screened using phylogenetic, cis-acting elements, GO annotation and PPI network analyses, which were used for qRT-PCR analysis with specific primers (Table S13).

An ABI 7500 Real-Time system (Applied Biosystems) was utilized to perform qRT-PCR. Each PCR mixture (20 μL) consisted of 0.4 μL of ROX Reference Dye II, 0.8 μL of upstream and downstream primers (10 μmol/L), 2 μL of cDNA template, 6 μL of double-distilled water (ddH_2_O) and 10 μL of 2 × SYBR (TB Green Premix Ex Taq II). The reaction conditions were as follows: 95 °C for 30 s, followed by 40 cycles of 95 °C for 5 s, 60 °C for 35 s, 95 °C for 15 s, 60 °C for 1 min, and 95 °C for 15 s. The 18S-RNA gene was used as the internal control and to normalize the expression data. The relative expression levels of the genes were calculated using the 2^−∆∆CT^ method. For each analysis, three biological replicates and three technical replicates were performed.

## 5. Conclusions

This is the first study concerning the genome-wide characterization of AP2/ERF superfamily TFs in *J. mandshurica*. Through extensive bioinformatics and gene expression profiling analysis, the conclusions of this study included (a) a total of 184 *JmAP2/ERF* genes identified in the *J. mandshurica* genome and were divided into five subfamilies through phylogenetic analysis; (b) the *JmAP2/ERF* superfamily was subject to gene duplication events and purifying selection pressure during evolution through gene structures and synteny analysis; (c) the expression profile under cold stress by qRT-PCR showed that 14 *JmAP2/ERF* genes were significantly upregulated, demonstrating their crucial functions in the resistance to cold stress; (d) *JmDREB-02*, *JmDREB-03*, *JmDREB-04*, *JmERF26*, and *JmERF27* were core TFs for enhancing the freezing tolerance of *J. mandshurica*, which can, thus, be used as candidate genes for genetic improvement of cold stress resistance. These results provide reliable information and a useful resource for better research into the biological roles of *JmAP2/ERFs* and plant genetic improvement.

## Figures and Tables

**Table 1:** Summary of the JmAP2/ERF superfamily TFs in the *J. mandshurica* genome.

Classification	Domains	Group	Number of Genes
RAV subfamily	AP2-B3		6
Soloist subfamily	AP2		4
AP2 subfamily	AP2	ANT	3
	AP2-AP2	AP2	24
	Total *JmAP2* genes		27
DREB subfamily	AP2	I	10
	AP2	II	11
	AP2	III	27
	AP2	IV	9
	Total *JmDREB* genes		57
ERF subfamily	AP2	V	19
	AP2	VI	8
	AP2	VII	5
	AP2	VIII	18
	AP2	IX	29
	AP2	X	8
	AP2	VI-L	3
	Total *JmERF* genes		90
Total *JmAP2/ERF* genes			184

## Data Availability

Data is contained within the article and Supplementary Materials.

## Supplementary Materials

The following supporting information can be downloaded at: https://www.mdpi.com/article/10.3390/ijms232315225/s1.
