# Supplementary material for: Genome-Wide Identification of AP2/ERF Superfamily Genes in Juglans mandshurica and Expression Analysis under Cold Stress
Source: Int J Mol Sci. 2022 Dec 3;23(23):15225. doi: 10.3390/ijms232315225 (PMC9736363; doi:10.3390/ijms232315225)
Supplement: Supplementary file 1 [file ijms-23-15225-s001.zip › Supplementary Figures.pdf]

Supplementary Figures

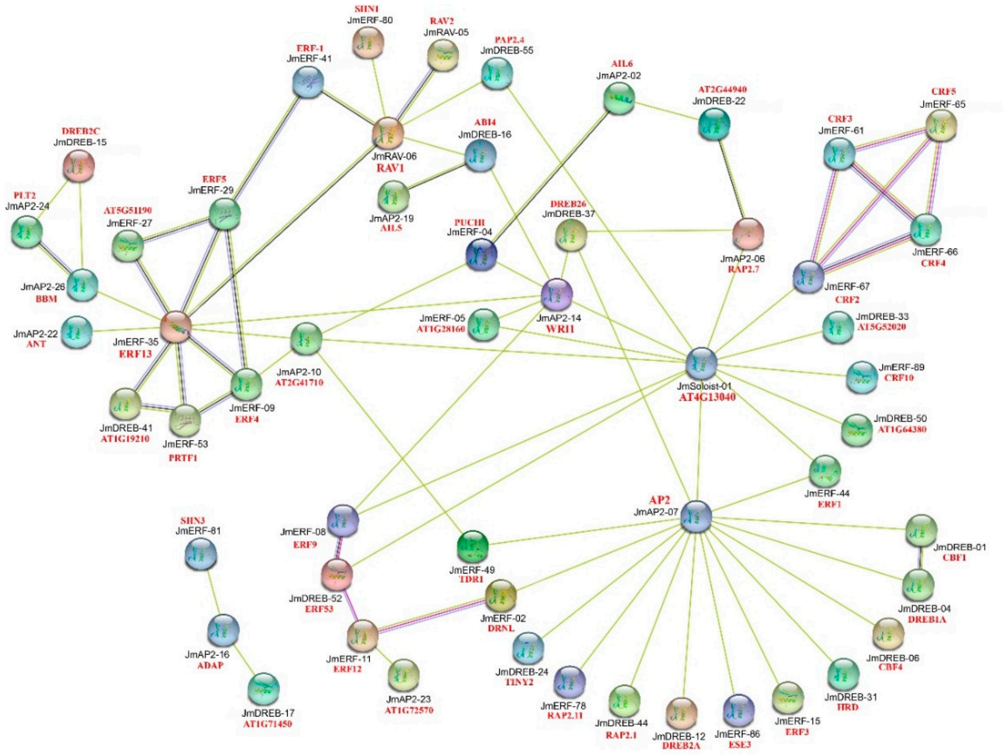

Figure S1. Protein-Protein interaction network of all *JmAP2/ERF* genes in *J. mandshurica*. Stronger associations are represented by thicker lines.

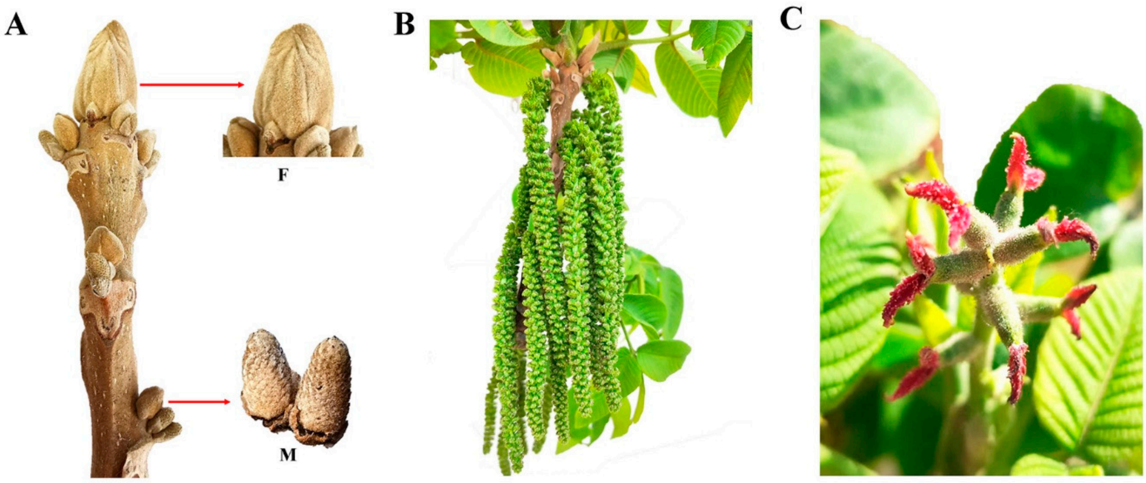

Figure S2. All experimental materials of *J. mandshurica* used in this study. The experimental materials are different tissues of *J. mandshurica*. (A), Current- year branches in winter. F is female flower buds. M is male flower buds. (B) Male flowers during the flowering period. (C), Female flowers during the flowering period.
